# Molluscum contagiosum palpébral: à propos d’un cas

**DOI:** 10.11604/pamj.2019.32.177.18418

**Published:** 2019-04-11

**Authors:** Moulay Omar Moustaine, Bouchra Allali, Loubna El Maaloum, Asmaa El Kettani, Khalid Zaghloul

**Affiliations:** 1 Service d’Ophtalmologie Pédiatrique, Hôpital 20 août, Centre Hospitalier Universitaire Ibn Rochd Casablanca, Casablanca, Maroc; 2 Service d’Ophtalmologie, Centre Hospitalier Provincial la Marche Verte, Boulmane-Missour, Maroc

**Keywords:** Tumeur palpébrale, Molluscum contagiosum, Molluscipoxvirus, Eyelid tumor, molluscum contagiosum, Molluscipoxvirus

## Abstract

Les *Molluscums contagiosum* (MC) sont des lésions cutanées bénignes causées par Molluscipoxvirus, ils affectent principalement les enfants et les adultes jeunes et intéressent surtout la peau et rarement les muqueuses. Le diagnostic clinique est facile, confirmé par l'analyse histologique de la lésion, cependant il n'existe pas de consensus concernant la prise en charge thérapeutique. La localisation palpébrale de MC est rare, elle pose un problème d'ordre diagnostic différentiel surtout lorsqu'elle est isolée et un problème thérapeutique vu la proximité du globe oculaire. Nous rapportons le cas d'une fille de 7 ans ayant présenté une lésion palpébrale isolée dont l'exérèse avec étude anatomopathologique a révélé un MC. A la lumière de cette observation nous décrivons les particularités cliniques, thérapeutiques, et évolutives de cette localisation rare du molluscum contagiosum.

## Abstract (en)

Molluscums contagiosum (MC) are benign skin lesions caused by Molluscipoxvirus, primarily affecting children and young adults. They manly involve the skin and rarely the mucous membranes. Clinical diagnosis is easy, confirmed by histological examination of the lesion. However there is no consensus regarding therapy. Eyelid molluscum contagiosum is rare, posing a problem of differential diagnosis especially when it is isolated as well as a therapeutic problem given the proximity of the eyeball. We report the case of a 7-year old girl with isolated eyelid lesion. The patient underwent lesion excision. Anatomopathological examination showed molluscum contagiosum. This study aims to describe the clinical, therapeutic and evolutionary features of this rare localization of molluscum contagiosum.

## Introduction

Le *Molluscums contagiosum* (MC) est une pathologie virale transmise par le Molluscipoxvirus, touchant la peau et plus rarement les muqueuses. Il est responsable de néoformations dermatologiques bénignes et survient le plus souvent chez les enfants, mais aussi chez les adolescents et les adultes jeunes chez qui il représente une infection sexuellement transmissible [1, 2]. Le diagnostic est clinique confirmé par l'analyse histologique de la lésion. La localisation palpébrale reste très rare et pose un problème d'ordre diagnostic différentiel et un autre de choix thérapeutique vu la proximité du globe oculaire. Nous rapportons le cas d'une fille de 7 ans ayant présenté un MC palpébral isolé et qui a bien évolué après exérèse chirurgicale par curetage minutieux. A la lumière de cette observation nous décrivons les particularités cliniques, thérapeutiques, et évolutives de cette localisation rare du MC.

## Patient et observation

**Figure 1:**
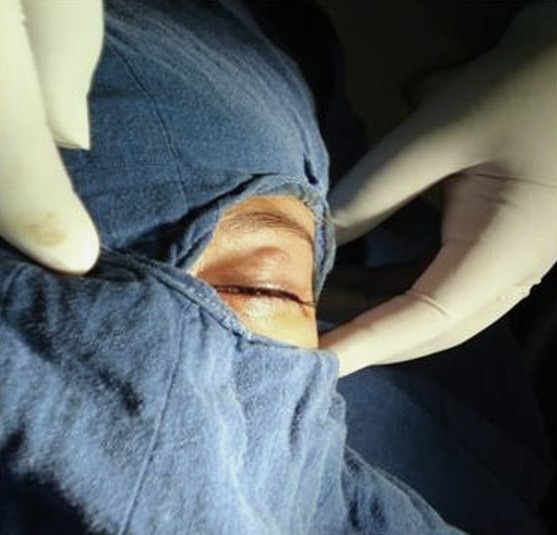
Aspect macroscopique de la lésion palpébrale évoquant un molluscum contagiosum palpébral isolé

**Figure 2:**
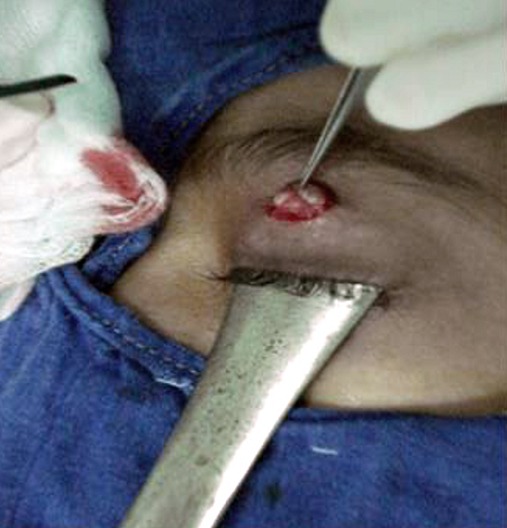
Exérèse chirurgicale du contenu par curettage

**Figure 3:**
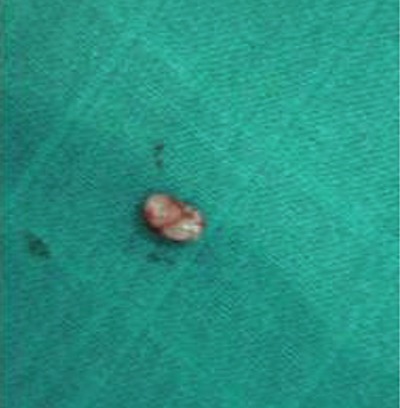
Noyant blanc du molluscum contagiosum après exérèse chirurgicale

Il s'agit de KH.M, fille de 7 ans suivie en pédiatrie pour un déficit immunitaire idiopathique, adressé en consultation ophtalmologique pour une lésion palpébrale gauche, apparue depuis quelques mois et augmentant progressivement de volume. À l´examen, on note une lésion nodulaire en relief, unique, ombiliqué au sommet, d'environ 10 mm de diamètre, indolore à surface irrégulière, localisée au niveau de la partie infra-sourcilière de la paupière supérieure gauche (Figure 1) et évoquant un molluscum contagiosum palpébral. Le reste de l'examen ophtalmologique est normal, notamment pas de conjonctivite folliculaire associée, l'examen dermatologique ne trouve pas de lésions similaires ailleurs et l'examen général est sans particularité. Une dissection chirurgicale faite sous sédation avec ablation complète de la lésion (Figure 2). Les suites postopératoires étaient simples avec une bonne cicatrisation cutanée (Figure 3). L'étude anatomopathologique de la lésion confirme le diagnostic de molluscum contagiosum. Le suivi à moyen terme n'a pas mis en évidence l'apparition de nouvelles lésions palpébrales ou cutanées.

## Discussion

**Figure 4:**
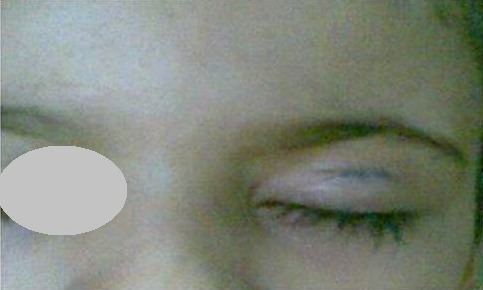
Aspect de la cicatrice cutané à J1 en post opératoire

Les MC sont des lésions cutanées bénignes transmissibles, causées par une infection virale due à un pox-virus (Molluscipoxvirus). Ils affectent principalement les enfants et les adultes jeunes et intéressent surtout la peau et rarement les muqueuses [1, 2]. Les lésions se présentent sous forme de papules perlées, ombiliquées et globuleuses, et peuvent se multiplier par auto inoculation favorisée par le grattage. La localisation palpébrale du MC est très rare, elle provoque outre une conjonctivite folliculaire ou une kératite superficielle, comme elle peut être complètement palpébrale isolée [3, 4]. Elle est plus fréquente avec des lésions particulièrement volumineuses et disséminées chez les immunodéprimés notamment VIH positifs [4, 5]. Cette localisation pose un problème de diagnostic différentiel avec certaines lésions palpébrales (verrue plane, adénome sébacé, chalazion). Elle peut aussi mimer des tumeurs palpébrales type histiocytose ou xanthogranulome. Chez les VIH positifs, il faudra toujours exclure un acanthome acantholytique [6]. Devant un doute diagnostique, la dermatoscopie permet une analyse clinique plus précise, tandis que l'étude histologique confirme le diagnostic en mettant en évidence des inclusions virales intra lésionnelles caractéristiques (corps de molluscum). Il s'agit d'inclusions denses, cerclées et éosinophiles particulièrement visibles dans la couche cornée [4, 7]. Malgré les nombreuses modalités thérapeutiques disponibles, il n´existe pas de consensus concernant la prise en charge du MC. En effet et compte-tenu le caractère autolimité des lésions et la possibilité de régression spontanée comme toutes les verrues en quelques mois, certains auteurs préfèrent une abstention thérapeutique avec surveillance régulière. D'autres par contre préconisent une attitude active afin de limiter l'auto-inoculation et la propagation des lésions sources parfois de complications (dermatite inflammatoire, surinfection) [8-10]. Le curetage minutieux de chaque lésion est l'attitude la plus fréquente, certes très efficace, mais douloureux et expose au risque important de récurrences [10-12]. Réalisé de façon rapide, il reste relativement bien toléré chez l'adulte, cependant chez l'enfant une application locale préalable d'une crème anesthésiante est nécessaire [13, 14]. Les autres thérapeutiques disponibles, à noter, la photothérapie dynamique, le laser à colorant pulsé, l'application de solutions et gels à base d'agents kératolytiques ou de huile essentielle (tea tree), ont permet des résultats encourageantes à travers divers publications, toutefois, à l'heure actuelle, il n'existe pas de protocole standard pour leur utilisation [2, 15-17]. Devant un MC palpébrale, cas de notre patiente, la proximité intime du globe oculaire et l'importance de la qualité cicatricielle dans cette région limite les choix thérapeutiques avec le risque d'effets indésirables et/ou de mauvaise cicatrice [12, 18]. Néanmoins une attitude active est toujours justifiable pour des raisons esthétiques et aussi pour prévenir la survenue de complications oculaires (conjonctivite, kératite). De ce fait l'exérèse chirurgicale par un curettage minutieux reste l'attitude recommandée en passant en priorité la qualité cicatricielle (Figure 4). Chez l'enfant très anxieux le recours à une sédation peut être nécessaire pour plus de sécurité [10, 17, 18].

## Conclusion

Le MC est une tumeur bénigne assez fréquente chez l´enfant, la localisation palpébrale est rare. Le diagnostic clinique est généralement aisé mais le choix thérapeutique est parfois problématique: récurrences, dissémination, terrain immunodéprimé. La localisation palpébrale justifie une attitude thérapeutique active avec exérèse chirurgicale par curetage minutieux en faisant passer en priorité la qualité cicatricielle.

## Conflits d’intérêts

Les auteurs ne déclarent aucun conflit d'intérêts.
